# Application of Field-of-View Optimized and Constrained Undistorted Single Shot (FOCUS) with Intravoxel Incoherent Motion (IVIM) in 3T in Locally Advanced Rectal Cancer

**DOI:** 10.1155/2021/5565902

**Published:** 2021-03-20

**Authors:** Yipeng Cheng, Huijie Jiang, Hui Wang, Qingchao Tang, Tianyi Liu

**Affiliations:** ^1^Department of Magnetic Resonance Imaging, The Second Affiliated Hospital of Harbin Medical University, Harbin, 150086 Heilongjiang, China; ^2^Department of Radiology, The Second Affiliated Hospital of Harbin Medical University, Harbin, 150086 Heilongjiang, China; ^3^Department of Colorectal Surgery, The Second Affiliated Hospital of Harbin Medical University, Harbin, 150086 Heilongjiang, China; ^4^Department of Pathology, The Second Affiliated Hospital of Harbin Medical University, Harbin, 150086 Heilongjiang, China

## Abstract

**Purpose:**

To evaluate the efficacy of field-of-view (FOV) optimized and constrained undistorted single shot (FOCUS) with IVIM in 3T MRI in the grading of patients with locally advanced rectal cancer.

**Methods:**

From January 1st to December 31st, 2019, patients with locally advanced rectal cancer were retrieved. FOCUS DWI and FOCUS IVIM were obtained. Apparent diffusion coefficient (ADC) and IVIM parameters including mean true diffusion coefficient (*D*), pseudodiffusion coefficient associated with blood flow (*D*∗), and perfusion fraction (*f*) of the tumor parenchyma and normal rectal wall, as well as the normalized tumor parameters by corresponding normal intestinal wall parameters (ADC_NOR_, *D*_NOR_, *D*∗_NOR_, and *f*_NOR_), were compared between the well/moderately differentiated and poorly differentiated groups by Student's *t*-test. The relationship between the above parameters and the histologic grade was analyzed using Spearman's correlation test, with the ROC curve generated.

**Results:**

Eighty-eight patients (aged 31 to 77 years old, mean = 56) were included for analysis. *D*_tumor_ and *f*_tumor_ were positively correlated with the tumor grade (*r* = 0.483, *p* < 0.001 and *r* = 0.610, *p* < 0.001, respectively). All the normalized parameters (ADC_NOR_, *D*_NOR_, *D*∗_NOR_, and *f*_NOR_) were positively correlated with the tumor grade (*r* = 0.267, *p* = 0.007; *r* = 0.564, *p* = 0.001; *r* = 0.414, *p* = 0.005; and *r* = 0.605, *p* < 0.001, respectively). The best discriminative parameter was the *f*_tumor_ value, and the area under the ROC curve was 0.927. With a cut-off value of 22.0%, *f*_tumor_ had a sensitivity of 88.9% and a specificity of 100%.

**Conclusion:**

FOCUS IVIM-derived parameters and normalized parameters are useful for predicting the histologic grade in rectal cancer patients.

## 1. Introduction

Rectal cancer is the 8th most common tumor and the 9th leading cause of cancer-related deaths worldwide [1]. Rectal cancer accounts for approximately 40% of cases of colorectal malignancies. Of note, it is the most common colorectal tumor in people < 50 years old, and the incidence in this population is on the rise [2].

MRI is the gold standard for the staging and assessment of treatment response of local rectal cancer lesions [3, 4]. Multimodality functional MRI also plays a very important role in the diagnosis, staging, and monitoring of the treatment efficacy of rectal cancer [5, 6]. Diffusion-weighted imaging (DWI) is a very common type of functional imaging, which can not only detect the site of rectal cancer but also noninvasively assess the morphological and functional changes in rectal cancer. However, conventional DWI suffers from susceptibility artifacts manifested as blurring or severe geometrical distortion [7, 8], which diminishes the diagnostic value of DWI. Field-of-view (FOV) optimized and constrained undistorted single shot (FOCUS) is an optimizing sequence of FOV that facilitates spatially selective excitation. This reduced field-of-view (rFOV) DWI sequence produces higher-quality images and higher repeatability in ADC measurement compared with the conventional full field-of-view (fFOV) DWI scans when applied to imaging thyroid gland [9], breast [10], colorectal tumor [11], and prostate cancers [12]. The DWI sequence is based on monoexponential fit function mode calculation, whereas ADC reflects the diffusion of water molecules in the living tissue and is also affected by the capillary perfusion effect of the capillary network and other micro- and macrofactors [13].

Intravoxel incoherent motion (IVIM) uses a biexponential mathematic model with multiple *b* values and noninvasively measures both the diffusion of free water molecules and the perfusion caused by microcirculation *in vivo*. The main parameters derived from the biexponential model are *D* (pure diffusion coefficient), *D*∗ (pseudodiffusion coefficient), and *f* (perfusion fraction) [14]. Many studies have shown that the IVIM parameters are important surrogate biomarkers to provide information about tissue physiology [15], with applications in evaluating liver fibrosis [16], transplant kidney function [17], and hepatocellular carcinoma grading [18], providing information on tumor microenvironment related to treatment effect, predicting tumor aggressiveness, and monitoring treatment response [19]. However, IVIM parameters still suffer from image artifacts, especially in gas-containing organs [20].

In this study, we investigated the feasibility of the FOCUS technique with IVIM in locally advanced rectal cancer patients imaged by 3.0T MRI and the relationship between FOCUS DWI, FOCUS IVIM parameters, and histologic grading of rectal cancer.

## 2. Materials and Methods

### 2.1. Patients

In accordance with the ethics guidelines for human research, this study was approved by the Institutional Ethics Review Board, and informed consent was obtained from all patients. We retrospectively identified patients who received FOCUS IVIM during a pilot study (data unpublished) using a 3T MR scanner from January 1st, 2019, to December 31st, 2019. The following inclusion criteria were all met. (1) Patients with endoscopic biopsy-proven rectal adenocarcinoma had surgical treatment within 2 weeks after MRI and had a pathological diagnosis of locally advanced rectal cancer. (2) Patients received no treatment for rectal cancer before this MRI examination. (3) There is no intrauterine device or metal foreign body in the pelvic cavity. (4) Patients had histopathologically proven nonmucinous adenocarcinoma [21]. Patients not meeting any one of the above criteria were excluded.

### 2.2. MRI Examination

All examinations were performed with a 3.0T system (GE Discovery MR750; GE Healthcare) using an 8-channel phased-array torso coil. Patients received bowel preparation, including a low-fiber diet one day before MRI, an 8-hour fast before MRI, and an enema with 500 ml of saline 30 min before MRI [22]. For all patients, the following five standard sequences were performed: (1) sagittal T2-weighted spin-echo sequence, (2) axial T1-weighted turbo spin-echo sequence, (3) axial T2-weighted short TI inversion recovery (STIR) sequence, (4) FOCUS DWI sequence, and (5) FOCUS IVIM sequence.

The FOCUS IVIM and DWI sequences scan range covered the largest area of the tumor and extended 2-3 cm beyond the distal border of the tumor. Spatial saturation bands were used to remove the signal from overlying fat and nearby tissues. The following 10 *b* values were used: 0, 50, 75, 100, 150, 200, 400, 600, 800, and 1000 s/mm^2^. The numbers of excitation (NEXs) were 1, 1, 2, 2, 2, 3, 3, 4, and 4, respectively. Other corresponding imaging parameters were as follows: repetition time/echo time (TR/TE): 2484 ms/65 ms; field of view: 240 mm × 96 mm; number of sections: 18-25; matrix size: 160 × 80; slice thickness: 5 mm; and interslice gap: 1 mm. For the FOCUS DWI sequence, two *b* values (0 and 1000 s/mm^2^) were used, and diffusion-weighted gradients were applied in three orthogonal directions. The remaining scan parameters were consistent with the FOCUS IVIM sequence.

IVIM parameter maps and ADC maps were generated and calculated using FuncTool (GE Healthcare 4.6). In the biexponential model, the IVIM parameters were calculated by the following equation: Sb/S0 = (1 − *f*)(−*bD*) + *f*exp(−*bD*∗) [14], where Sb is the signal intensity in the pixel with the diffusion gradient, S0 is the signal intensity in the pixel without a diffusion gradient, and *b* is the diffusion sensitivity determined by the difference between *b* and b0, which, in this case, includes all *b* values (0, 50, 75, 100, 150, 200, 400, 600, 800, and 1000 s/mm^2^) fitted that equation. *D* is the true diffusion as reflected by pure molecular diffusion, *D*∗ is the pseudodiffusion coefficient related to perfusion, and *f* is the fractional perfusion related to microcirculation.

ADC maps were calculated on a pixel-by-pixel basis and reconstructed according to the following equation: ADC = −(In[S1] − In[S0])/(b1 − b0), where S1 is the signal intensity of a voxel after application of the diffusion gradient, and S0 is the signal magnitude without diffusion gradients applied (*b* = 0 s/mm^2^, b1 = 1000 s/mm^2^).

### 2.3. Image Analysis

From FOCUS IVIM, diffusion images were obtained with all *b* values and IVIM parameter maps (*D* map, *D*∗ map, and *f* map). In the original diffusion images, the diffusion of water molecules was limited in the tumor parenchyma, but it was significantly higher in the normal intestinal wall. All regions of interest (ROIs) were manually delineated and contoured in the original diffusion images. One radiologist with 11 years of experience, who was kept blind to the histologic grade of each case, reviewed and evaluated original diffusion images of FOCUS IVIM and then selected the largest area of the tumor mass parenchyma (used for tumor parameters) and the distal end of the normal rectal wall (used for rectal wall parameters, at a distance from the edge of the tumor of over half the circumference of the rectum, without any signs of infiltration). A single representative ROI was traced manually along the margin of the tumor or the normal rectal wall; then, the position of the ROI was automatically placed on IVIM parametric maps. To reduce measurement errors, 3 ROIs (on DWI representing low *b* values *b* = 150 s/mm^2^, medium *b* values *b* = 600 s/mm^2^, and high *b* values *b* = 1000 s/mm^2^, respectively) were manually drawn at the selected level. To further minimize bias, each area was measured 3 times and the average parameter values were calculated. The ROI excluded macroscopic necrosis, visible vessels, and gut contents, and ROIs were delineated carefully to discard the areas with movement artifacts or image degradation owing to signal loss or voxel misalignments.

For each ROI, the mean values of *D*, *f*, and *D*∗ were calculated from the corresponding parameter maps.

The normalized parameter values were defined as the ratio between the parameter_tumor_ and the parameter_rectal wall_, using the following equation:

(1) $$\begin{array}{c} \text{Normalized parameter values}=\frac{\;{\text{parameter}}_{\text{tumor}}}{{\text{parameter}}_{\text{rectal wall}}}. \end{array}$$

The ADC value obtained and normalization in the FOCUS DWI sequence are consistent with the IVIM analysis.

### 2.4. Histopathology Evaluation

Surgical pathology specimens of all patients were evaluated by a pathologist with 11 years of experience in the gastrointestinal pathologic diagnosis, who was kept blind to the original pathology report as well as the MRI data. The histopathologic type and tumor differentiation (including well-differentiated, moderately differentiated, or poorly differentiated) were recorded. If the rectal carcinoma had mixed degrees of differentiation, the tumor was defined using the worst grade. Well-differentiated and moderately differentiated cases were combined into a single group to simplify statistical analysis. Cases of mucinous adenocarcinoma were excluded, which was characterized by abundant extracellular mucin that constitutes more than 50% of the tumor volume.

### 2.5. Statistical Analysis

All statistical analyses were performed using SPSS version 26.0 (Chicago, IL, USA). Discrete data were shown as the number of cases and analyzed using the *χ*^2^ test. Continuous data were expressed as mean ± standard deviation (SD) and were analyzed using Student's *t*-test or the Mann-Whitney *U* test if the assumption of homogeneity of variance between the groups was violated. A two-tailed *p* < 0.05 was considered significantly different.

Histopathology was considered the standard reference for the statistical evaluation of FOCUS DWI, FOCUS IVIM parameters, and normalized parameters. ADC, *D*, *D*∗, *f*, ADC_NOR_, *D*_NOR_, *D*∗_NOR_, and *f*_NOR_ of the rectal tumor were compared. The fitness of the numeric dataset to normal distribution was determined by the Kolmogorov-Smirnov test. The correlation between the FOCUS DWI, FOCUS IVIM parameters, or normalized parameters and the rectal tumor histologic grade were analyzed with Spearman's correlation test. Receiver operating characteristic (ROC) analysis was performed to evaluate the diagnostic value of parameters for detection of the tumor grade and to determine the sensitivity and specificity of the tests.

## 3. Results

**Figure 1:**
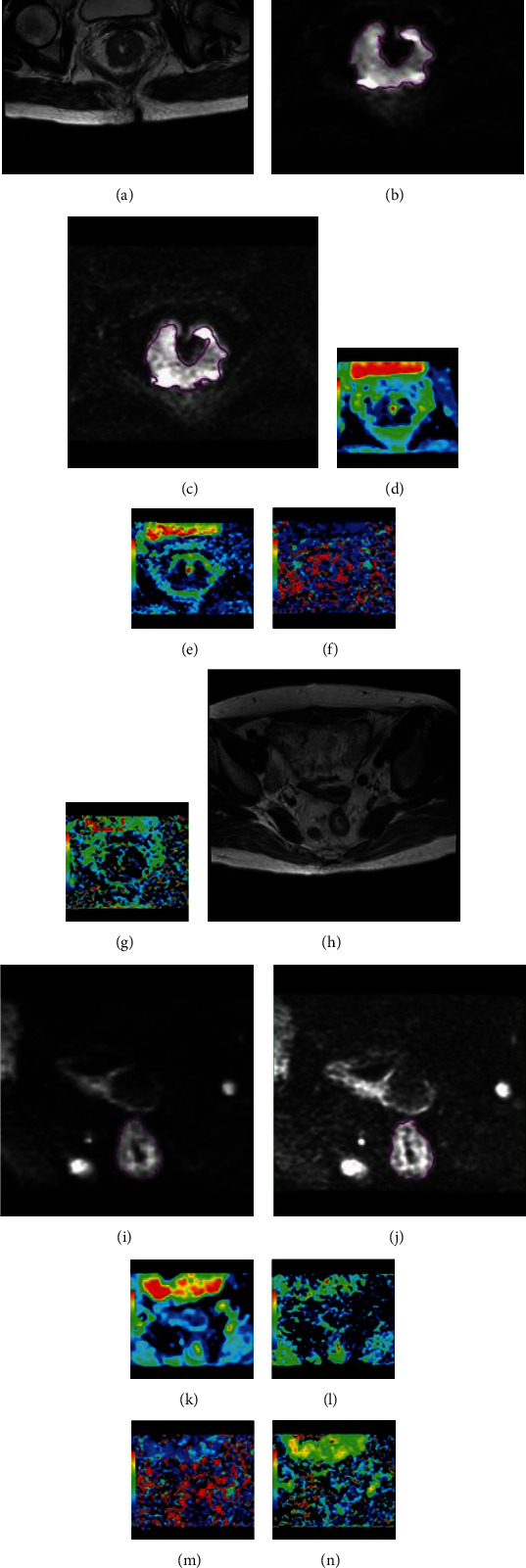
A 63-year-old woman with rectal cancer histopathologically diagnosed as poorly differentiated and stage T3 N2 cM0. (a) Axial T2-weighted MRI shows masses in the rectal wall. (b, c) Shows hyperintensity on FOCUS DWI and the diffusion trace image with a *b* value of 1000 s/mm^2^ of FOCUS IVIM. (d) The corresponding ADC, (e) pure diffusion coefficient *D*, (f) pseudodiffusion coefficient *D*∗, and (g) perfusion-related fraction *f* maps of the tumor. (h) Axial T2-weighted MRI shows the normal rectal wall. (i, j) Shows isointensity on FOCUS DWI and the diffusion trace image with a *b* value of 1000 s/mm^2^ of FOCUS IVIM. (k) The corresponding ADC, (i) pure diffusion coefficient *D*, (m) pseudodiffusion coefficient *D*∗, and (n) perfusion-related fraction *f* maps of the normal rectal wall.

**Figure 2:**
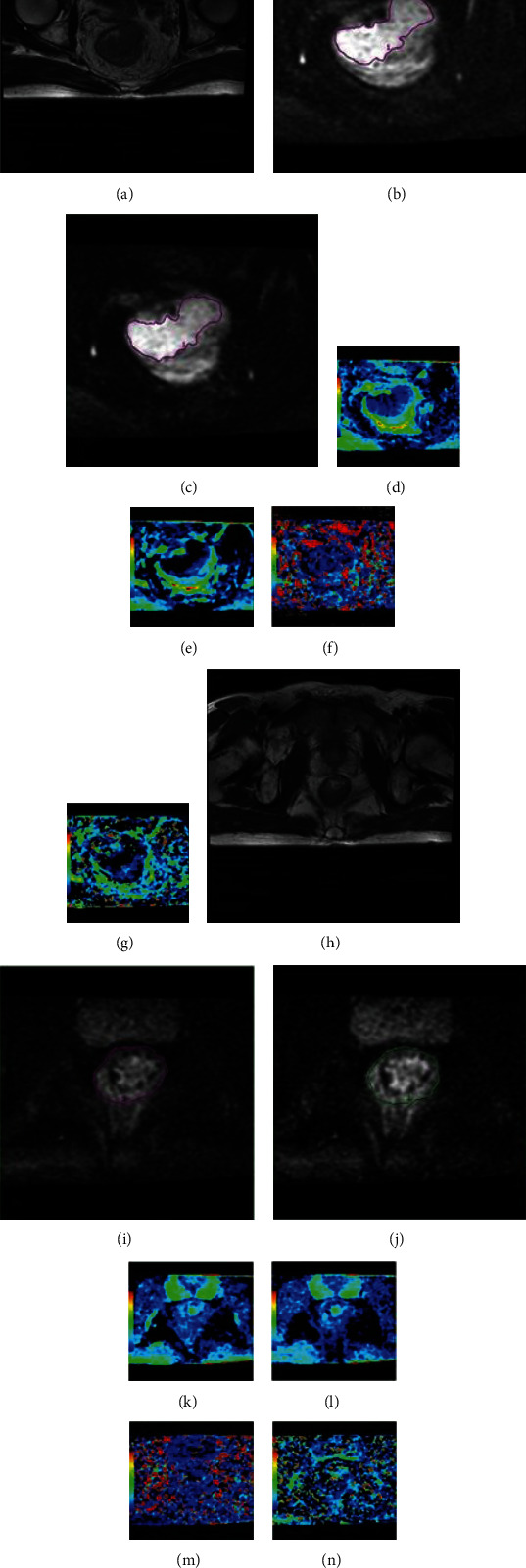
A 56-year-old woman with rectal cancer histopathologically diagnosed as well/moderately differentiated and stage T3 N0 cM0. (a) Axial T2-weighted MRI shows masses in the rectal wall. (b, c) Shows hyperintensity on FOCUS DWI and the diffusion trace image with a *b* value of 1000 s/mm^2^ of FOCUS IVIM. (d) The corresponding ADC, (e) pure diffusion coefficient *D*, (f) pseudodiffusion coefficient *D*∗, and (g) perfusion-related fraction *f* maps of the tumor. (h) Axial T2-weighted MRI shows the normal rectal wall. (i, j) Shows isointensity on FOCUS DWI and the diffusion trace image with a *b* value of 1000 s/mm^2^ of FOCUS IVIM. (k) The corresponding ADC, (i) pure diffusion coefficient *D*, (m) pseudodiffusion coefficient *D*∗, and (n) perfusion-related fraction *f* maps of the normal rectal wall.

**Figure 3:**
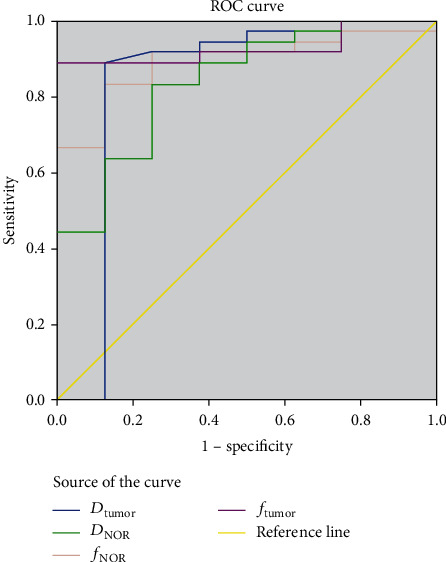
ROC curve of discriminative parameters.

Out of the 436 cases with locally advanced rectal cancer who received MRI scans, 88 patients had FOCUS IVIM (Figure S1) and were included in the study as detailed in Table 1. There were no differences in age, gender, or BMI between the well/moderately differentiated tumor group and the poorly differentiated tumor group. Quantitative analyses of the FOCUS DWI, FOCUS IVIM parameters, and normalized parameters are shown in Table 2. The correlation of the histologic grade with ADC and IVIM parameters is shown in Table 3. The poorly differentiated tumor group (Figure 1) had significantly lower *D*_tumor_, *f*_tumor_, *D*_NOR_, and *f*_NOR_ values than the well/moderately differentiated tumor group (Figure 2) (*p* = 0.011, *p* < 0.001, *p* = 0.001, and *p* < 0.001, respectively, Table 2). *D*_tumor_ and *f*_tumor_ values were positively correlated with the tumor grade (*r* = 0.483, *p* < 0.001 and *r* = 0.610, *p* < 0.001, respectively). No significant difference in the parameters of the intestinal wall of the distal tumor (ADC_rectal wall_, *D*_rectal wall_, *D*∗_rectal wall_, and *f*_rectal wall_) was found between the well/moderately differentiated tumors and the poorly differentiated tumors (*p* = 0.388, *p* = 0.378, *p* = 0.150, and *p* = 0.153, respectively). All the normalized parameters (ADC_NOR_, *D*_NOR_, *D*∗_NOR_, and *f*_NOR_) were positively correlated with the tumor grade (*r* = 0.267, *p* = 0.007; *r* = 0.564, *p* = 0.001; *r* = 0.414, *p* = 0.005; and *r* = 0.605, *p* < 0.001, respectively). The best discriminative parameter was *f*_tumor_, and the area under the ROC curve was 0.927. With a cut-off value of 22.0%, *f*_tumor_ had a sensitivity of 88.9% and a specificity of 100% (Table 4 and Figure 3).

## 4. Discussion

This study demonstrated that FOCUS IVIM technology produced analyzable DWI and parameter data, which can be used for quantitative research. Certain FOCUS IVIM parameters and normalized parameters were positively correlated with the grade of rectal cancer.

The international guidelines recommended MRI as a crucial method for primary staging and restaging of rectal carcinoma after chemotherapy and radiation therapy (CRT) [5]. Functional MRI, such as functional DWI and IVIM, is an important supplement to conventional DWI, which can indicate biological behavior, helping oncologists predict potential treatment effects and assess prognosis. However, the rectum is a particularly challenging area for DWI and IVIM using 3T MRI. Intestinal peristalsis, gas in the rectum, and intestinal contents can produce susceptible artifacts and increase geometric deformation, which affects the accuracy of measured parameters. These inevitable distortions and artifacts are caused by slow traversal through the k-space line and the narrow bandwidth.

Several techniques have been developed for higher-resolution DWI that decrease the effective encoded FOV to achieve the desired in-plane resolution while limiting distortion [23, 24]. FOCUS is an optimized sequence that facilitates spatially selective excitation [25], in which a 2-dimensional spatially selective echo-planar radiofrequency excitation pulse and a 180° refocusing pulse can reduce the FOV in the phase-encode direction, reduce the number of baselines required for k-space filling, shorten the readout time and echo time (TE) [26], and consequently acquire high-spatial resolution images with less distortion [12]. The FOCUS technique has been applied by other investigators for DWI of prostate cancer [12], endometrial cancer [27], and colorectal cancer [11]. However, there is no published literature about the application of the FOCUS technique with IVIM.

In our study, we found that ADC could not differentiate carcinomas of different histologic grades, which contradicts the results of Curvo-Semedo et al., who reported that poorly differentiated rectal carcinoma had a lower ADC value [28]. The differences may be due to the influence of tumor microcirculation perfusion effects [29] and due to the influence of image artifacts, distortions, or partial volume effects that may have existed in previous studies.

In our study, *D*_tumor_ was shown to be significantly different between different grades and was moderately correlated with tumor differentiation. These findings were consistent with the study by Liu et al. [30], which showed that water molecules move less freely in poorly differentiated tumors due to the increased cellularity with hydrophobic membrane integrity, extracellular tortuosity, and disorganization. *D*_tumor_ was useful for distinguishing good and poor responders after chemotherapy and radiation therapy in rectal cancer [31]. Whereas in other studies, *D*_tumor_ could not clarify the diversity among variously differentiated carcinomas [32, 33]. This controversy may be caused by the tumor boundary distortions and artifacts, signal-to-noise ratio, or partial volume effect. The FOCUS IVIM technique may provide a good quality image and comparably accurate location of lesions on IVIM images for parameter measurement.

The derived perfusion-related parameter *D*∗ has been correlated with microvessel density (MVD) in tumor specimens stained by anti-CD31 [34], and *D*∗ has the potential to serve as a noninvasive approach for monitoring endostatin-induced tumor vascular normalization [35]. *D*∗ actually reflects the blood flow, whose velocity and vascular geometry may be affected by the poor functional nature of neovessels [36]. Therefore, the measurement of IVIM-derived *D*∗ values in different tumors produced a broad range, which were consistent with the different vascularity of different tumor types [32, 37]. Our study also obtained the same results using FOCUS IVIM technology, reflecting that the selection and implementation of IVIM mathematical models are still challenging.

Our study demonstrated a significantly lower *f*_tumor_ for poorly differentiated rectal carcinoma and a significant positive correlation between *f*_tumor_ and differentiation, which were also consistent with previous studies [32, 33, 38]. The underlying mechanism may be that in poorly differentiated tumors, the chaotic organization and structural instability of tumor vasculature could lead to a lower perfusion fraction. We also found that *f*_tumor_ had the strongest correlation among all parameters, and according to the ROC curve, *f*_tumor_ was the best discriminative parameter for the determination of the degree of rectal cancer differentiation.

This study also investigated the relationship between the normalized parameters (ADC_NOR_, *D*_NOR_, *D*∗_NOR_, and *f*_NOR_) and the differentiation of rectal cancer. Although there was no significant difference in the parameters of the normal intestinal wall of the distal tumor (ADC_rectal wall_, *D*_rectal wall_, *D*∗_rectal wall_, and *f*_rectal wall_), poorly differentiated tumors had significantly lower *D*_NOR_ and *f*_NOR_ values than well/moderately differentiated ones. Moreover, all the normalized parameters (ADC_NOR_, *D*_NOR_, *D*∗_NOR_, and *f*_NOR_) were positively correlated with the tumor grade, suggesting a beneficial role of normalization using the normal rectal wall as self-control.

## 5. Conclusion

IVIM images with the FOCUS technique provide optimal signal-to-noise resolution and can produce reliable parameters for qualitative analysis. IVIM parameters show a strong correlation with the tumor grade and differentiation and have the potential to provide clinically useful information about diffusion and perfusion parameters which may be helpful in predicting tumor aggressiveness and prognosis.

## Figures and Tables

**Table 1:** Clinical characteristics of rectal cancer patients in the well/moderately differentiated group and poorly differentiated group.

Factors	Well/moderately differentiated (*n* = 62)	Poorly differentiated (*n* = 26)	*p* value
Age (years)^#^	55.8 ± 11.5	55.9 ± 10.4	0.97^∗^
Male	34	12	0.46^∗∗^
BMI^#^	24.3 ± 3.8	24.2 ± 2.7	0.89^∗^

^#^Mean ± SD. ^∗^*t*-test. ^∗∗^*χ*^2^.

**Table 2:** Results of quantitative FOCUS DWI, FOCUS IVIM analysis, and corresponding normalized parameters.

Parameters	Well/moderately differentiated (mean ± SD, *n* = 62)	Poorly differentiated (mean ± SD, *n* = 26)	*p* valve
ADC_tumor_ (×10^–3^ mm^2^/s)	1.08 ± 0.14	1.01 ± 0.20	0.204
*D*_tumor_ (×10^–3^ mm^2^/s)	0.93 ± 0.17	0.68 ± 0.24	0.011
*D*∗_tumor_ (×10^–3^ mm^2^/s)	51.79 ± 19.94	47.00 ± 19.41	0.541
*f*_tumor_ (%)	28.85 ± 6.26	18.88 ± 2.17	<0.001^∗^
ADC_rectal wall_ (×10^–3^ mm^2^/s)	1.47 ± 0.19	1.55 ± 0.29	0.388
*D*_rectal wall_ (×10^–3^ mm^2^/s)	1.07 ± 0.18	1.01 ± 0.23	0.378
*D*∗_rectal wall_ (×10^–3^ mm^2^/s)	37.18 ± 22.68	50.90 ± 29.43	0.150
*f*_rectal wall_ (%)	39.38 ± 5.85	36.14 ± 4.79	0.153
ADC_NOR_	0.75 ± 0.12	0.67 ± 0.16	0.141
*D*_NOR_	0.82 ± 0.15	0.62 ± 0.13	0.001
*D*∗_NOR_	1.82 ± 1.12	2.02 ± 3.13	0.861^∗^
*f*_NOR_	0.74 ± 0.14	0.53 ± 0.08	<0.001

^∗^By the Mann-Whitney *U* test. *p* < 0.05 indicates a statistically significant difference.

**Table 3:** Correlation of the histologic grade with ADC, IVIM parameters, and corresponding normalized parameters.

Parameters	*r*	*p*
ADC_tumor_	0.193	0.209
*D*_tumor_	0.483	<0.001
*D*∗_tumor_	0.194	0.207
*f*_tumor_	0.610	<0.001
ADC_NOR_	0.267	0.007
*D*_NOR_	0.564	0.001
*D*∗_NOR_	0.414	0.005
*f*_NOR_	0.605	<0.001

**Table 4:** ROC analysis of IVIM parameters.

Parameters	AUC	95% confidence interval	Cut-off value	Sensitivity (%)	Specificity (%)
*D*_tumor_ (×10^–3^ mm^2^/s)	0.839	0.613~1.000	0.664	88.9	87.5
*f*_tumor_ (%)	0.927	0.849~1.000	22	88.9	100.0
*D*_NOR_	0.840	0.693~0.987	0.684	88.3	75.0
*f*_NOR_	0.892	0.791~0.993	0.610	88.3	87.5

## Data Availability

Data and study protocol are available from the corresponding author on reasonable request.

## Supplementary Materials

Supplementary Materials Figure S1: flow chart of the patient screening process.
