# Supplementary figures and images for: Application of Field-of-View Optimized and Constrained Undistorted Single Shot (FOCUS) with Intravoxel Incoherent Motion (IVIM) in 3T in Locally Advanced Rectal Cancer
Source: Dis Markers. 2021 Mar 20;2021:5565902. doi: 10.1155/2021/5565902 (PMC8055408; doi:10.1155/2021/5565902)

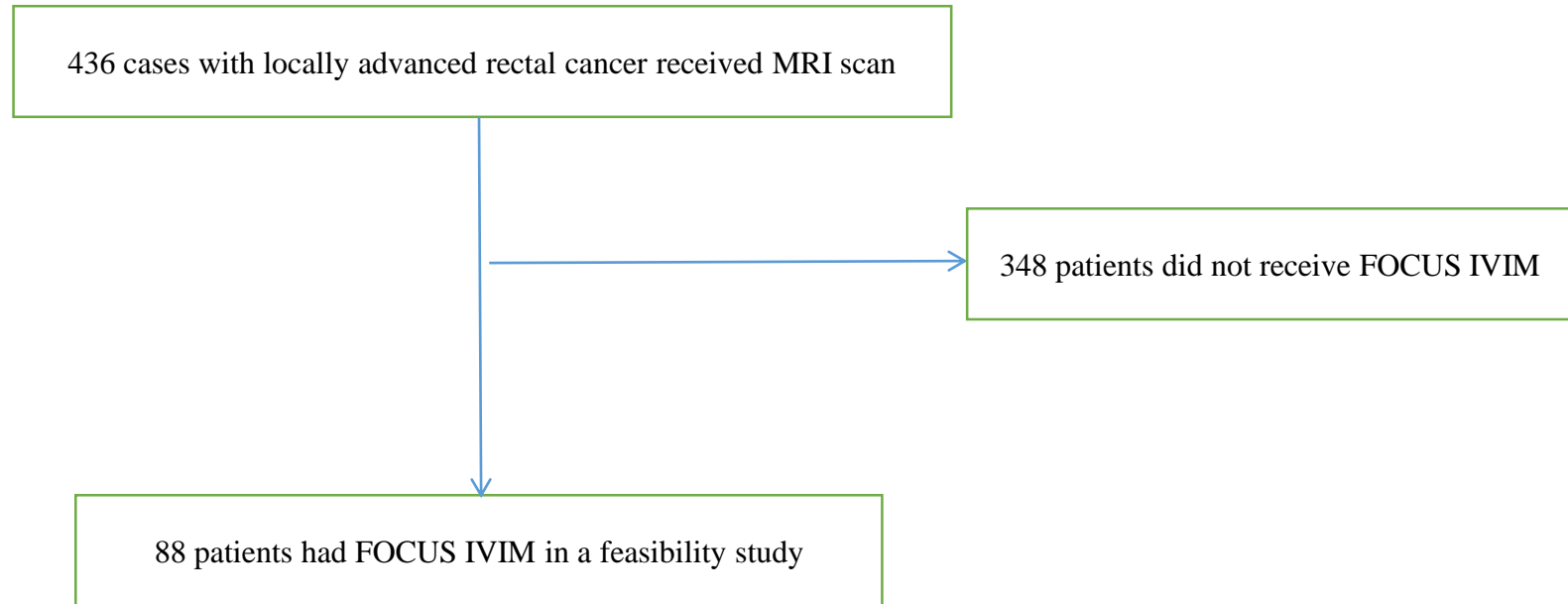

Supplement: Supplementary Materials — Figure S1: flow chart of the patient screening process. [file 5565902.f1.pdf]
